# Associations between childhood trauma, intolerance of uncertainty, and symptom severity in obsessive-compulsive disorder

**DOI:** 10.3389/fpsyt.2026.1871808

**Published:** 2026-06-24

**Authors:** Hasan Ünver, Safiye Zeynep Tatlı, Tayfun Öz, İbrahim Hakkı Karakuş, Mehmet Rıdvan Varlı, Aybüke Demir, Kübra Özcan Çetin, İbrahim Gündoğmuş

**Affiliations:** 1 Department of Psychiatry, Ankara Etlik City Hospital, Ankara, Türkiye; 2 Department of Psychiatry, Başakşehir Çam and Sakura City Hospital, Istanbul, Türkiye; 3 Department of Psychiatry, School of Medicine, Istanbul Nişantaşı University, Istanbul, Türkiye

**Keywords:** childhood trauma, intolerance of uncertainty, mediation analysis, obsessive-compulsive disorder (OCD), symptom severity

## Abstract

**Background:**

Childhood trauma (CT) has been associated with obsessive-compulsive disorder (OCD), but its relationship with obsessive-compulsive symptom (OCS) severity remains inconsistent. Intolerance of uncertainty (IU) may represent one of the cognitive processes underlying this association. The present study aimed to examine differences in CT and IU between patients with OCD and healthy controls (HCs), and to test whether IU mediates the relationship between CT and OCS severity.

**Methods:**

This study included 82 patients with OCD and 82 healthy controls (HCs) matched on age and sex. CT was assessed using the Childhood Trauma Questionnaire-33 (CTQ-33), IU using the Intolerance of Uncertainty Scale–Short Form (IUS-12), and OCS severity using the Yale-Brown Obsessive-Compulsive Scale (Y-BOCS).

**Results:**

Patients with OCD had significantly higher scores than HCs on all CTQ-33 subscales and on IU measures. In particular, the patient group showed higher IUS-12 total scores than the HC group (39.30 ± 10.42 vs. 32.11 ± 8.62, p < 0.001), with higher prospective anxiety (22.11 ± 5.13 vs. 20.11 ± 4.59, p = 0.009) and inhibitory anxiety scores (17.19 ± 5.99 vs. 12.00 ± 4.82, p < 0.001). Within the patient group, physical abuse was the only CT dimension significantly associated with total Y-BOCS scores (r = 0.248, p = 0.025), whereas IU was positively associated with symptom severity (IUS-12 total: r = 0.346, p = 0.001). Path analysis showed that CT was associated with IU (β = 0.238, p = 0.023), IU was associated with OCS severity (β = 0.329, p = 0.007), and the direct effect of CT on OCS severity was no longer significant after IU was included in the model (c′ = 0.209, p = 0.093), supporting partial mediation.

**Conclusion:**

CT appears to be elevated in patients with OCD, although its association with symptom severity is not uniform across trauma dimensions. IU may represent an important cognitive mechanism linking CT to OCS severity. These findings suggest that assessing and addressing IU may contribute to more individualized clinical approaches in OCD.

## Introduction

Although childhood trauma (CT) has been consistently associated with adult psychopathology, its relationship with obsessive-compulsive symptom (OCS) severity in obsessive-compulsive disorder (OCD) remains unclear. Evidence from clinical samples suggests that CT is more common in patients with OCD than in healthy controls (HCs) and may be associated with greater symptom severity (1–3). However, findings across studies have been inconsistent, varying according to trauma subtype, symptom dimension, and sample characteristics, with generally small effect sizes (4). Together, these findings suggest that the relationship between CT and OCS severity is heterogeneous and may not be explained by trauma exposure alone (5, 6).

One possible explanation for this heterogeneity is that the association between CT and OCS severity may operate through cognitive processes that influence how individuals perceive and respond to uncertainty. Among these processes, intolerance of uncertainty (IU) appears particularly relevant. IU refers to a dispositional tendency to respond negatively to uncertain situations at cognitive, emotional, and behavioral levels (7, 8), and has been identified as a transdiagnostic construct across emotional disorders (9, 10). Within OCD, IU has been closely linked to doubt, the need for certainty, and repetitive behaviors aimed at reducing uncertainty (10, 11). Because OCS often involve attempts to manage or neutralize uncertainty, IU may provide a useful framework for understanding how CT relates to symptom severity.

CT may be especially relevant to IU because adverse early-life experiences often involve unpredictability, lack of control, and heightened threat sensitivity (12, 13). Such experiences may contribute to long-term difficulties in tolerating uncertainty, which in turn may affect the severity of obsessive-compulsive symptoms. Previous studies have separately examined CT, IU, and OCD-related symptoms, but studies integrating these variables within a single analytical framework remain limited. In particular, the potential mediating role of IU in the relationship between CT and OCS severity has received relatively little attention (7, 11).

Therefore, the present study aimed to examine CT and IU in patients with OCD compared with HCs, and to test whether IU mediates the relationship between CT and OCS severity within the patient group. We hypothesized that patients with OCD would report higher levels of CT and IU than HCs. We further hypothesized that greater CT would be associated with greater OCS severity in the patient group, and that this association would be partially mediated by IU.

## Methods

### Study sample

The study included a total of 82 patients diagnosed with OCD according to the Diagnostic and Statistical Manual of Mental Disorders, Fifth Edition (DSM-5), who presented to the Psychiatry Outpatient Clinic of Etlik City Hospital between November 2024 and December 2025. All diagnoses were established using the Structured Clinical Interview for DSM-5 Disorders – Clinician Version (SCID-5/CV), administered by a trained psychiatrist. Participants were recruited consecutively from the outpatient clinic. At the time of assessment, all patients were receiving routine psychiatric treatment. The study also included 82 HCs matched to the patient group on age and sex at the group level. The absence of current psychiatric disorders in the control group was confirmed using the SCID-5/CV.

Inclusion criteria for the patient group were: (1) being 18–65 years of age, (2) having a DSM-5 diagnosis of OCD confirmed with the SCID-5/CV, confirmed with the SCID-5/CV, and (3) having at least a primary school level of education. Inclusion criteria for the control group were: (1) being 18–65 years of age, (2) having no psychiatric diagnosis according to DSM-5 criteria, as assessed with the SCID-5/CV, and (3) having no major neurological disorder.

Exclusion criteria for both groups included major neurological disorders, psychiatric conditions affecting cognitive functioning, sensory impairments interfering with assessment, intellectual disability, current alcohol or substance use disorder, unstable or severe medical conditions that could interfere with psychiatric assessment, pregnancy or postpartum status, and a history of significant head trauma (loss of consciousness >30 minutes). Stable chronic medical conditions under treatment and regular clinical follow-up were not considered exclusion criteria.

A total of 94 patients were initially screened for participation. Of these, 3 were excluded due to intellectual disability, 2 due to postpartum status, and 7 due to incomplete scale data. After these exclusions, the final patient sample consisted of 82 individuals with OCD.

The study was approved by the Clinical Research Ethics Committee of Etlik City Hospital (approval date; 27.11.2024, decision number: AEŞH-BADEK-2024-1066). Written informed consent was obtained from all participants prior to study participation.

### Procedure

After providing written informed consent, participants were assessed in a structured sequence. First, eligibility was evaluated according to the inclusion and exclusion criteria. In the patient group, the diagnosis of OCD was confirmed using the SCID-5/CV, and in the control group, the absence of any current psychiatric disorder was verified using the same interview. Following the diagnostic interview, OCS severity was evaluated using the Yale-Brown Obsessive-Compulsive Scale (Y-BOCS). Both the SCID-5/CV and the Y-BOCS were administered by a trained psychiatrist. After completion of the clinical interview process, participants completed the self-report measures, including the Sociodemographic Data Form, the Childhood Trauma Questionnaire-33 (CTQ-33), and the Intolerance of Uncertainty Scale–Short Form (IUS-12). Clinical and sociodemographic information was obtained from participant self-report and clinical records.

### Assessment instruments

#### Sociodemographic data form

The Sociodemographic Data Form is a structured form developed by the researchers to collect sociodemographic data, including age, sex, marital status, education level, income level, employment status, and place of residence, as well as clinical characteristics, including presence of chronic illness, smoking status, alcohol use, history of suicide attempts, family history of psychiatric disorders and suicide, and duration of illness. Data were obtained from participant self-report and clinical records.

#### Structured clinical interview for DSM-5 disorders – clinician version

The SCID-5/CV is a semi-structured interview developed to systematically and reliably assess psychiatric diagnoses based on DSM-5 criteria (14). The Turkish validity and reliability study of the SCID-5/CV was conducted by Elbir et al. (15). In the present study, the SCID-5/CV was administered by a trained psychiatrist to confirm the diagnosis of OCD in the patient group and to rule out any psychiatric disorder in the control group.

#### Yale-brown obsessive-compulsive scale

The Y-BOCS is a clinician-administered, semi-structured instrument developed to assess the type and severity of OCS (16). It consists of 10 items, with five assessing obsessions and five assessing compulsions. Each item is rated on a 0–4 scale, yielding a total score ranging from 0 to 40, with higher scores indicating greater symptom severity. The Turkish validity and reliability study was conducted by Karamustafalıoğlu et al. (17). The Y-BOCS was administered by a trained psychiatrist.

#### Childhood trauma questionnaire-33

The CTQ-33 is an expanded self-report instrument derived from the Childhood Trauma Questionnaire and is used to assess emotional, physical, and sexual abuse, as well as emotional and physical neglect during childhood and adolescence. In addition to these five domains, the CTQ-33 includes an overprotection/overcontrol (OP-OC) subscale (18, 19). The instrument consists of 33 items rated on a 5-point Likert scale, with higher scores indicating greater exposure to CT (19). For group comparisons and correlation analyses, CTQ-33 subscale scores were used. For the mediation analysis, the CTQ-33 total score was used as an overall indicator of CT.

#### Intolerance of uncertainty scale – short form (IUS-12)

The IUS-12 is a self-report instrument used to assess levels of IU (20). The scale consists of 12 items and includes two subdimensions: prospective anxiety and inhibitory anxiety. Items are rated on a 5-point Likert scale, with higher scores indicating greater IU. The Turkish validity and reliability study of the scale was conducted by Sarıçam et al. (21).

### Statistical analysis

Statistical analyses were performed using SPSS version 20.0. Descriptive statistics were presented as frequencies and percentages for categorical variables and as means ± standard deviations for continuous variables. The normality of continuous variables was assessed using the Kolmogorov-Smirnov and Shapiro-Wilk tests. Group comparisons between the patient and HC groups for continuous variables presented in **Table 1** were conducted using independent-samples t tests, whereas categorical variables in **Table 2** were analyzed using chi-square tests. Effect sizes for continuous between-group comparisons were calculated using Cohen’s d. Within the patient group, relationships among CTQ-33 subscale scores, IUS-12 scores, and OCS severity were examined using Pearson correlation analyses. To examine the potential mediating role of IU in the relationship between CT and OCS severity, a path analysis with observed variables was conducted using AMOS. In this model, the CTQ-33 total score was entered as the predictor, the IUS-12 total score as the mediator, and the Y-BOCS total score as the outcome variable. Total, direct, and indirect effects were estimated. The significance of the indirect effect was evaluated using bootstrap 95% confidence intervals. An indirect effect was considered statistically significant when the confidence interval did not include zero. Model fit was evaluated using standard fit indices, including the chi-square statistic (CMIN), the chi-square/degrees of freedom ratio (CMIN/df), the Goodness-of-Fit Index (GFI), the Comparative Fit Index (CFI), and the Incremental Fit Index (IFI).

**Table 1:** Comparison of childhood trauma and intolerance of uncertainty between patients and healthy controls.

Variable	Patients mean (SD)	Healthy controls mean (SD)	t	Df	p	Cohen's d
Y-BOCS						
*Obsession*	11.62 (3.34)	—				
*Compulsion*	11.46 (3.78)	—				
*Total*	23.08 (6.75)	—				
CTQ-33						
*Emotional Abuse*	9.40 (4.63)	6.48 (2.00)	5.226	162	**<0.001**	0.819
*Physical Abuse*	6.77 (3.26)	5.45 (1.58)	3.312	162	**0.001**	0.515
*Physical Neglect*	8.56 (6.34)	6.25 (2.51)	3.058	162	**0.003**	0.479
*Emotional Neglect*	12.75 (6.36)	8.65 (3.71)	5.051	162	**<0.001**	0.788
*Sexual Abuse*	6.59 (3.03)	5.19 (0.82)	4.045	162	**<0.001**	0.631
*OP-OC*	10.63 (4.54)	8.61 (3.22)	3.290	162	**0.001**	0.513
IUS-12						
*Prospective Anxiety*	22.11 (5.13)	20.11 (4.59)	2.630	162	**0.009**	0.411
*Inhibitory Anxiety*	17.19 (5.99)	12.00 (4.82)	6.116	162	**<0.001**	0.955
*Total*	39.30 (10.42)	32.11 (8.62)	4.815	162	**<0.001**	0.752

CTQ-33, Childhood Trauma Questionnaire-33; OP-OC, Overprotection/Overcontrol; IUS-12, Intolerance of Uncertainty Scale–Short Form; Y-BOCS, Yale-Brown Obsessive-Compulsive Scale. All between-group comparisons presented in this table were conducted using the independent-samples t test. Cohen's d values are reported as effect size estimates for between-group differences. Bold values indicate statistically significant group differences (p < .05).

**Table 2:** Sociodemographic and clinical characteristics of the patients and healthy controls.

Variable	Patients (n=82)	Healthy controls (n=82)	χ² / t	df	p
Age (years), mean (SD)	30.82 (10.93)	30.57 (7.02)	0.178	162	0.859
Education, n (%)			18.811	3	<0.001
* Primary*	3 (3.7)	0 (0)			
* Secondary*	16 (19.5)	2 (2.4)			
* High school*	24 (29.3)	42 (51.2)			
* University*	39 (47.6)	38 (46.3)			
Gender, n (%)			2.712	1	0.100
* Female*	59 (72.0)	49 (59.8)			
* Male*	23 (28.0)	33 (40.2)			
Income status, n (%)			0.989	2	0.610
* Low*	11 (13.4)	10 (12.2)			
* Middle*	24 (29.3)	19 (23.2)			
* High*	47 (57.3)	53 (64.6)			
Marital status, n (%)			1.800	2	0.407
* Married*	42 (51.2)	38 (46.3)			
* Single*	37 (45.1)	37 (45.1)			
* Divorced*	3 (3.7)	7 (8.5)			
Employment status, n (%)			9.809	1	0.002
Unemployed	48 (58.5)	28 (34.1)			
Employed	34 (41.5)	54 (65.9)			
Place of residence, n (%)			0.689	1	0.406
* City*	66 (80.5)	70 (85.4)			
* District*	16 (19.5)	12 (14.6)			
Chronic illness, n (%)			2.313	1	0.128
* No*	66 (80.5)	73 (89.0)			
* Yes*	16 (19.5)	9 (11.0)			
Smoking, n (%)			0.451	1	0.502
* No*	58 (70.7)	54 (65.9)			
* Yes*	24 (29.3)	28 (34.1)			
Alcohol use, n (%)			4.071	1	**0.044**
* No*	68 (82.9)	57 (69.5)			
* Yes*	14 (17.1)	25 (30.5)			
History of suicide attempts, n (%)			6.926	1	**0.008**
* No*	69 (84.1)	79 (96.3)			
* Yes*	13 (15.9)	3 (3.7)			
Family history of psychiatric disorders, n (%)			7.317	1	**0.007**
* No*	54 (65.9)	69 (84.1)			
* Yes*	28 (34.1)	13 (15.9)			
Family history of suicide, n (%)			2.768	1	**0.096**
* No*	77 (93.9)	81 (98.8)			
* Yes*	5 (6.1)	1 (1.2)			
Duration of illness (years), mean (SD)	8.34 (7.88)	—			

Values are presented as mean (SD) or n (%). χ²: chi-square test; t: independent samples t-test. Bold values indicate statistically significant group differences (p < 0.05).

### Statistical significance was set at p < 0.05.

An *a priori* power analysis was conducted using G*Power 3.1. Because the primary analyses involved comparisons between the patient and HC groups on continuous variables, the calculation was based on an independent-samples t test. Assuming a medium effect size (Cohen’s d = 0.50), a two-tailed alpha level of 0.05, and a statistical power of 0.80, the minimum required total sample size was 128 participants (64 per group). The final sample consisted of 164 participants, including 82 patients with OCD and 82 HCs, which exceeded the minimum required sample size.

## Results

### Sample characteristics

A total of 94 patients were screened for eligibility. After excluding 3 patients due to intellectual disability, 2 due to postpartum status, and 7 due to incomplete scale data, the final sample consisted of 82 patients with OCD and 82 HCs. The sociodemographic and clinical characteristics of the patient and HC groups are presented in **Table 2**. There were no significant differences between the groups in terms of age (p = 0.859), gender distribution (p = 0.100), income status (p = 0.610), marital status (p = 0.407), place of residence (p = 0.406), presence of chronic illness (p = 0.128), smoking status (p = 0.502), or family history of suicide (p = 0.096). However, significant differences were observed in education level (χ² = 18.811, p < 0.001) and employment status (χ² = 9.809, p = 0.002), with a higher proportion of unemployed individuals in the patient group. Alcohol use was significantly more frequent in the HC group (χ² = 4.071, p = 0.044). In contrast, the patient group reported significantly higher rates of suicide attempts (χ² = 6.926, p = 0.008) and family history of psychiatric disorders (χ² = 7.317, p = 0.007). In the patient group, the mean duration of illness was 8.34 ± 7.88 years. The most commonly reported initial obsession was contamination (54.9%), followed by doubt (29.3%) and intrusive thoughts (15.9%). Similarly, contamination remained the most prevalent current obsession (51.2%), followed by doubt (29.3%), intrusive thoughts (17.1%), and symmetry (2.4%).

### Group comparisons in childhood trauma and intolerance of uncertainty

Comparisons of CT and IU between the patient and HC groups are presented in **Table 1**. The mean Y-BOCS total score in the patient group was 23.08 ± 6.75.

Regarding CT, patients with OCD reported significantly higher scores than HCs across all CTQ-33 subscales, including emotional abuse (p < 0.001), physical abuse (p = 0.001), physical neglect (p = 0.003), emotional neglect (p < 0.001), and sexual abuse (p < 0.001). OP-OC scores were also significantly higher in the patient group (p = 0.001).

Similarly, IU scores were significantly elevated in the patient group compared with the HC group. Both prospective anxiety (p = 0.009) and inhibitory anxiety (p < 0.001) subdimensions, as well as the total IUS-12 score (p < 0.001), were higher among patients with OCD. The difference was more pronounced for inhibitory anxiety compared to prospective anxiety. According to Cohen’s d values, the magnitude of the between-group differences ranged from small-to-moderate to large, with inhibitory anxiety showing the largest effect size.

### Correlation analyses within the patient group

Pearson correlation analyses among CT, IU, and OCS severity within the patient group are presented in **Table 3**. CTQ-33 subscales were positively and significantly intercorrelated, indicating substantial overlap among different types of adverse experiences.

**Table 3:** Correlations among childhood trauma, intolerance of uncertainty, and obsessive-compulsive symptom severity in the patient group.

		CTQ-33 Emotional Abuse	CTQ-33 Physical Abuse	CTQ-33 Physical Neglect	CTQ-33 Emotional Neglect	CTQ-33 Sexual Abuse	CTQ-33 OP-OC	IUS-12 Prospective Anxiety	IUS-12 Inhibitory Anxiety	IUS-12 Total	Y-BOCS Obsession	Y-BOCS Compulsion	Y-BOCS Total
CTQ-33 Emotional Abuse	r	—											
	p	—											
CTQ-33 Physical Abuse	r	**0.624**	**—**										
	p	**< .001**	**—**										
CTQ-33 Physical Neglect	r	**0.298**	**0.354**	**—**									
	p	**< .001**	**< .001**	**—**									
CTQ-33 Emotional Neglect	r	**0.559**	**0.434**	**0.370**	**—**								
	p	**< .001**	**< .001**	**< .001**	**—**								
CTQ-33 Sexual Abuse	r	**0.204**	**0.286**	**0.266**	**0.200**	—							
	p	**0.009**	**< .001**	**< .001**	**0.010**	—							
CTQ-33 OP-OC	r	**0.491**	**0.295**	**0.203**	**0.462**	**0.210**	—						
	p	**< .001**	**< .001**	**0.009**	**< .001**	**0.007**	—						
IUS-12 Prospective Anxiety	r	**0.167**	0.101	-0.088	0.126	0.080	**0.193**	—					
	p	**0.033**	0.198	0.263	0.108	0.309	**0.013**	—					
IUS-12 Inhibitory Anxiety	r	**0.194**	0.118	0.043	**0.212**	**0.259**	**0.167**	**0.726**	—				
	p	**0.013**	0.132	0.588	**0.006**	**< .001**	**0.033**	**< .001**	—				
IUS-12 Total	r	**0.196**	0.119	-0.018	**0.186**	**0.192**	**0.192**	**0.914**	**0.942**	—			
	p	**0.012**	0.130	0.823	**0.017**	**0.014**	**0.014**	**< .001**	**< .001**	—			
Y-BOCS Obsession	r	0.134	0.182	-0.078	0.110	0.101	-0.057	**0.295**	**0.312**	**0.325**	—		
	p	0.229	0.104	0.484	0.327	0.369	0.610	**0.007**	**0.004**	**0.003**	—		
Y-BOCS Compulsion	r	0.150	**0.283**	0.029	0.199	0.110	0.029	**0.256**	**0.358**	**0.332**	**0.798**	—	
	p	0.179	**0.011**	0.795	0.073	0.324	0.793	**0.020**	**< .001**	**0.002**	**< .001**	—	
Y-BOCS Total	r	0.150	**0.248**	-0.022	0.166	0.111	-0.012	**0.290**	**0.355**	**0.346**	**0.941**	**0.955**	—
	p	0.178	**0.025**	0.841	0.137	0.319	0.916	**0.008**	**0.001**	**0.001**	**< .001**	**< .001**	—

CTQ-33, Childhood Trauma Questionnaire-33; IUS-12, Intolerance of Uncertainty Scale; Y-BOCS, Yale-Brown Obsessive-Compulsive Scale; OP-OC: Overprotection/Overcontrol; r = Pearson correlation coefficient. Bold values indicate statistically significant correlations (p < .05).

Weak but statistically significant positive correlations were observed between certain CTQ-33 dimensions and total IUS-12 scores. Specifically, emotional abuse (r = 0.196, p = 0.012), emotional neglect (r = 0.186, p = 0.017), sexual abuse (r = 0.192, p = 0.014), and OP-OC (r = 0.192, p = 0.014) were positively associated with IU.

IUS-12 total scores showed consistent and moderate positive correlations with OCS severity. Total IUS-12 scores were significantly associated with total Y-BOCS scores (r = 0.346, p = 0.001). Both prospective anxiety (r = 0.290, p = 0.008) and inhibitory anxiety (r = 0.355, p = 0.001) were positively correlated with symptom severity, with inhibitory anxiety showing the highest correlation with symptom severity.

In contrast, most CTQ-33 subscales were not significantly associated with OCS severity. Only physical abuse showed a weak but significant positive correlation with total Y-BOCS scores (r = 0.248, p = 0.025).

### Mediation analysis

**Figure 1:**
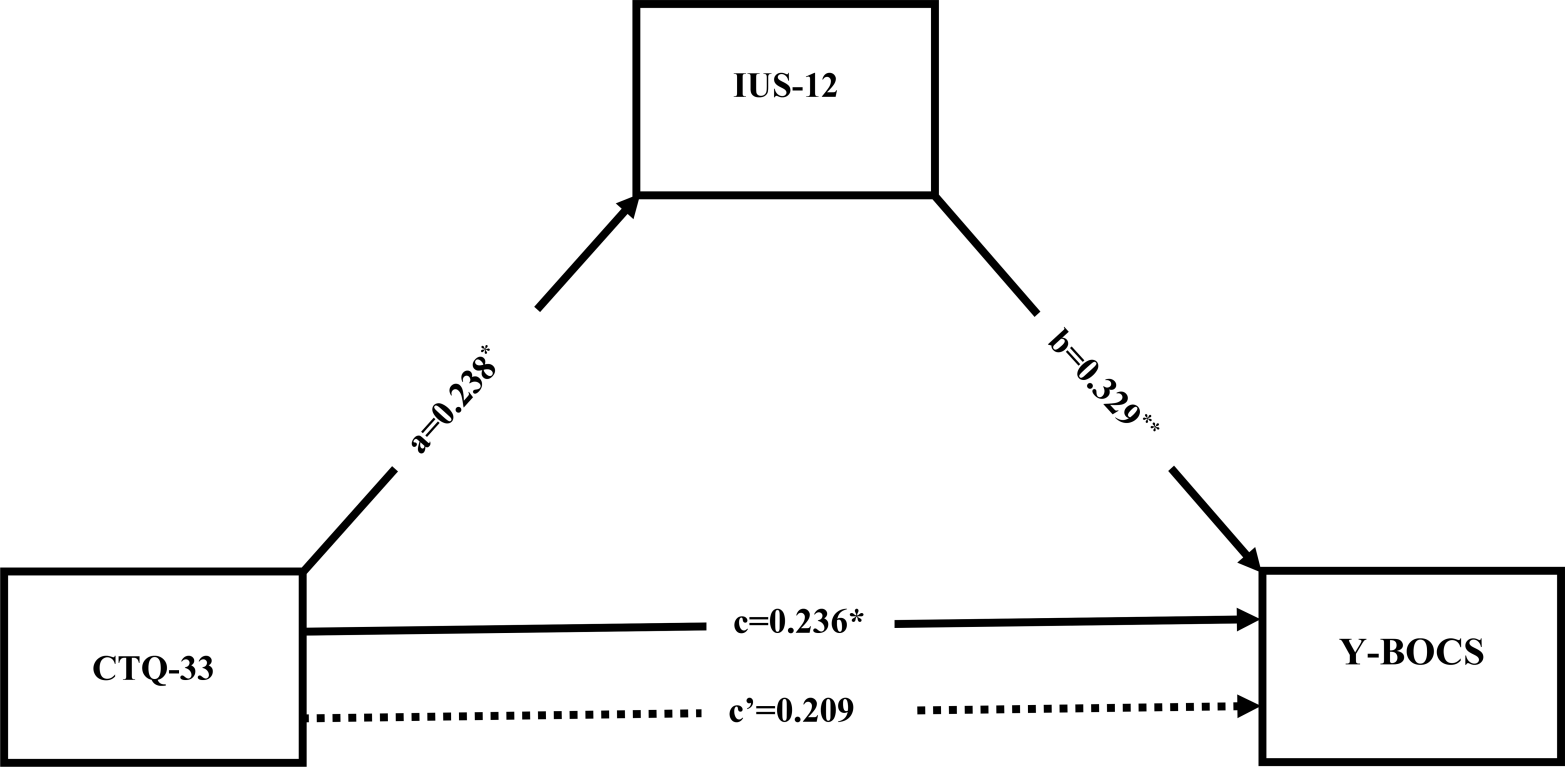
The mediating role of intolerance of uncertainty in the relationship between childhood trauma and obsessive–compulsive symptom severity in the patient group. Standardized coefficients are presented. c = total effect; c′ = direct effect. a = 0.238 (p = 0.023), b = 0.329 (p = 0.007), c = 0.236 (p = 0.029), and c′ = 0.209 (p = 0.093). *p < 0.05, **p < 0.01. Model fit indices indicated an acceptable fit (CMIN/df = 2.037, GFI = 0.928, CFI = 0.931, IFI = 0.934). CTQ-33, Childhood Trauma Questionnaire-33; IUS-12, Intolerance of Uncertainty Scale–Short Form; Y-BOCS, Yale-Brown Obsessive-Compulsive Scale.

The mediating role of IU in the relationship between CT and OCS severity is presented in **Figure 1**. In this model, the CTQ-33 total score was entered as the predictor, the IUS-12 total score as the mediator, and the Y-BOCS total score as the outcome variable. The CTQ-33 total score was significantly associated with OCS severity in the total effect model (c path: β = 0.236, p = 0.029; B = 0.284, SE = 0.183, t = 1.772). However, when IU was included in the model, the direct effect was no longer statistically significant (c′ path: β = 0.209, p = 0.093; B = 0.244, SE = 0.145, t = 1.680). The CTQ-33 total score was significantly associated with the IUS-12 total score (a path: β = 0.238, p = 0.023; B = 0.361, SE = 0.159, t = 2.267), and the IUS-12 total score was significantly associated with OCS severity (b path: β = 0.329, p = 0.007; B = 0.254, SE = 0.093, t = 2.718). The indirect effect was statistically significant, indicating that IU partially mediated the relationship between CT and OCS severity. Model fit indices indicated an acceptable fit (CMIN = 64.231, df = 32, p = 0.001; CMIN/df = 2.037, GFI = 0.928, CFI = 0.931, IFI = 0.934).

## Discussion

The present study yielded several findings, with the most important being that IU appears to play a mediating role in the relationship between CT and OCS severity. In the mediation model, CT was significantly associated with OCS severity in the total effect model; however, this direct relationship was no longer statistically significant when IU was included, while the paths from CT to IU and from IU to symptom severity remained significant. This pattern suggests that the association between CT and OCS severity may operate, at least in part, through cognitive responses to uncertainty rather than through a direct and uniform effect of trauma alone. In this respect, the present findings extend previous literature by suggesting that IU may represent one of the mechanisms through which adverse early experiences become clinically relevant in OCD.

This finding is conceptually meaningful. CT often involves chronic exposure to unpredictability, uncontrollability, and heightened threat sensitivity, all of which may shape later expectations about the environment and increase vulnerability to uncertainty-related distress (12, 13). IU, in turn, has been consistently described as a transdiagnostic cognitive disposition involving negative cognitive, emotional, and behavioral responses to uncertain situations (7–11). Within OCD, IU has been closely linked to doubt, checking, reassurance seeking, and repeated attempts to reduce ambiguity or prevent feared outcomes (11, 22–24). From this perspective, the present mediation finding supports the view that CT may contribute to obsessive-compulsive symptomatology not only as a distal risk factor, but also through its association with maladaptive cognitive styles, particularly difficulty tolerating uncertainty.

The correlational findings further support this interpretation. Within the patient group, IU was positively associated with CT and with OCS severity. Although the associations between IU and CT dimensions were small, they were statistically significant for emotional abuse, emotional neglect, sexual abuse, and overprotection/overcontrol (OP-OC). This pattern suggests that emotionally salient or controlling early experiences may be particularly relevant to later difficulties in tolerating uncertainty. In line with this, previous research has suggested that early adverse experiences, especially emotional forms of trauma, may be associated with elevated IU in adulthood (7). The association with OP-OC may also be noteworthy, as overprotective or overcontrolling early environments may foster reduced tolerance for ambiguity and heightened dependence on certainty, potentially contributing to later obsessive-compulsive vulnerability.

IU was also significantly associated with OCS severity, and this association appeared more robust than the direct associations between most CT dimensions and symptom severity. Both prospective anxiety and inhibitory anxiety were positively related to Y-BOCS scores, with inhibitory anxiety showing the stronger association. In addition, both IU dimensions were significantly associated with obsession and compulsion scores, suggesting that IU is relevant to both major components of OCD symptomatology. Clinically, this is important because it indicates that uncertainty-related cognitive processes may be involved not only in the intrusive and doubt-laden nature of obsessions, but also in the behavioral attempts to reduce uncertainty that characterize compulsions. This interpretation is consistent with prior work showing that IU is strongly linked to obsessive-compulsive symptoms and may show differential associations across OCD-related dimensions and emotional disorders more broadly (11, 25, 26).

A second important finding was that both CT and IU levels were significantly higher in the patient group than in the HC group. This result is consistent with earlier literature showing that CT is more common in OCD than in non-clinical populations (1–3), and also supports prior findings identifying IU as a clinically relevant construct in OCD (11, 25). Together, these group differences provide an important background for interpreting the mediation finding. That is, patients with OCD not only reported more adverse childhood experiences, but also showed greater difficulty tolerating uncertainty, which may represent one of the pathways linking early adversity to symptom expression. In addition, the patient group showed a significantly higher rate of suicide attempts than the HC group. This variable was included as part of the broader clinical characterization of the sample rather than as a primary study outcome. The observed difference may reflect the greater overall clinical burden of the patient group; however, because suicide attempt history was not included in the main correlational or mediation analyses, this finding should be interpreted cautiously.

At the same time, the direct associations between CT and OCS severity within the patient group were limited and heterogeneous. Among CT dimensions, only physical abuse showed a weak but significant association with total Y-BOCS scores. Notably, physical abuse was also positively associated with compulsion scores, suggesting that this trauma dimension may be more closely related to compulsive manifestations than to obsessive symptoms alone. More broadly, however, the absence of significant associations for most CT subdimensions indicates that CT is unlikely to influence symptom severity in a simple or uniform way. This is consistent with previous studies suggesting that the relationship between CT and OCD severity varies across trauma types, symptom dimensions, and clinical samples (2–4). Systematic reviews have similarly emphasized that this relationship is heterogeneous and generally modest in magnitude (2–4). Some studies have reported greater symptom severity and poorer treatment outcome in patients with childhood maltreatment histories (27), whereas others have linked specific trauma types to particular symptom profiles rather than to overall severity (28). The present findings fit this literature and suggest that trauma may be better understood as a background vulnerability whose clinical expression depends partly on intermediary cognitive processes such as IU.

From a clinical perspective, these findings highlight the potential importance of assessing IU in patients with OCD, particularly in those with a history of CT. Trauma-related experiences may not always be directly reflected in symptom severity scores, yet they may still contribute to the maintenance of symptoms through maladaptive ways of coping with uncertainty. This suggests that addressing IU may be especially relevant in case formulation and treatment planning. Interventions targeting IU may help reduce the impact of doubt, indecisiveness, and repeated certainty-seeking behaviors, and may therefore offer added clinical value for a subgroup of patients whose symptom presentation is shaped by both trauma-related vulnerability and uncertainty-related cognitive processes (10).

Several limitations should be considered when interpreting these findings. First, the cross-sectional design precludes causal inferences regarding the relationships among CT, IU, and OCS severity. Accordingly, the mediation model should be interpreted as statistically consistent with an indirect pathway rather than as proof of a causal mechanism. Second, CT was assessed using self-report, which may have introduced recall bias. Third, potentially relevant clinical variables, such as comorbid anxiety and depressive symptoms, were not controlled for in the analyses. In addition, the groups differed in several sociodemographic and clinical characteristics, including education level, employment status, alcohol use, history of suicide attempts, and family history of psychiatric disorders, which may have influenced the observed group differences. Despite these limitations, the study also has several strengths, including the use of a clinically diagnosed OCD sample, the inclusion of a HC group, and the simultaneous examination of CT, IU, and OCS severity within a single analytical framework.

In summary, the present findings suggest that the most clinically meaningful link between CT and OCS severity may not be a direct one, but rather one that operates through difficulty tolerating uncertainty. IU may therefore represent an important cognitive mechanism connecting adverse early experiences to OCS expression. Emphasizing this pathway may help refine both theoretical understanding and individualized clinical approaches in OCD.

## Conclusion

In conclusion, the present findings suggest that the most clinically meaningful association between CT and OCS severity may not be a direct one, but rather one that operates through IU. Although CT was elevated in patients with OCD and showed limited direct associations with symptom severity, IU was associated with both CT and OCS severity and appeared to partially mediate this relationship. These findings support the view that IU may represent an important cognitive mechanism linking adverse early experiences to OCS expression. Clinically, this suggests that assessing and addressing IU may help refine individualized case formulation and treatment planning in OCD, particularly for patients with a history of CT. Given the cross-sectional design, these findings should be interpreted cautiously and cannot be considered causal.

## Data Availability

The raw data supporting the conclusions of this article will be made available by the authors, without undue reservation.
